# Breakfast in Denmark. Prevalence of Consumption, Intake of Foods, Nutrients and Dietary Quality. A Study from the International Breakfast Research Initiative

**DOI:** 10.3390/nu10081085

**Published:** 2018-08-14

**Authors:** Sisse Fagt, Jeppe Matthiessen, Camilla Thyregod, Karsten Kørup, Anja Biltoft-Jensen

**Affiliations:** 1 National Food Institute, Division of Risk Assessment and Nutrition, Technical University of Denmark, 2800 Copenhagen, Denmark; jmat@food.dtu.dk (J.M.); karko@food.dtu.dk (K.K.); apbj@food.dtu.dk (A.B.-J.); 2 DTU Compute, Department of Applied Mathematics and Computer Science, Technical University of Denmark, 2800 Copenhagen, Demark; camt@dtu.dk

**Keywords:** Breakfast, dietary intake, foods, nutrition, dietary quality, NRF 9.3, index

## Abstract

Breakfast is considered by many to be the most important meal of the day. This study examined the intake of nutrients and foods at breakfast among Danes and the relation to the overall dietary quality. Data were derived from the Danish National Survey on Diet and Physical Activity 2011–2013, a cross-sectional national food consumption study. A total of 3680 participants aged 6–75 years were included in the analyses of breakfast consumption. The Nutrient Rich Food Index 9.3 method was used to examine the overall dietary quality of the diet. The intake of nutrients and foods at breakfast were compared across dietary quality score tertiles by ANCOVA adjusted for energy and socio economic status. Breakfast was eaten frequently by children and adults and contributed with 18–20% of total energy intake. Breakfast was relatively high in dietary fibre, B vitamins, calcium and magnesium and low in added sugar, total fat, sodium, vitamin A and D. A decrease in the intake of added sugar, total fat and saturated fat and an increase in the intake of dietary fibre and most micronutrients were seen across tertiles of dietary quality scores. Commonly consumed foods provided at breakfast in Denmark included bread, breakfast cereals and dairy products as well as water, coffee and juice, while intakes of fruits, vegetables, cakes and soft drinks were low.

## 1. Introduction

It is often stated that breakfast is the most important meal of the day and this has been integrated into the public mindset [1,2]. Recommendations on the intake of meals, foods, and nutrients in Denmark are based on the Nordic Nutrition Recommendations and the Danish food-based dietary guidelines (FBDG). The Danish FBDG do not mention when and how much breakfast should be eaten. Previously, the Nordic Nutrition Recommendations (NNR 2004) suggested that breakfast should account for 20–25% of the total energy intake [3]. However, the latest version of the NNR does not include recommendations on the proportion of energy from breakfast [4]. Thus, there is no official Danish or Nordic recommendation for energy and macro- and micronutrient intakes at breakfast.

The Danish Veterinary and Food Administration have a few recommendations for the timing of breakfast consumption. It is emphasized that skipping breakfast can have a negative influence on cognitive capacity and children, in particular, can have difficulty in reaching an adequate intake of daily nutrients [5]. The Danish FBDG only include a minor emphasis on specific meal based recommendations. One example is through the public-private partnership with the aim of increasing the intake of whole grain in the Danish population [6]. The Danish Whole Grain Partnership emphasizes that breakfast (and dinner) presents the greatest potential for an increased intake of whole grain [6,7].

The typical meal pattern in Denmark consists of three main meals and two to three in-between-meals. Breakfast is typically based on the intake of bread or breakfast cereals and dairy products. The intake of fruits, vegetables, fish, meat, and cakes are seldom consumed at breakfast. Water, milk, coffee, and juice are typically consumed at breakfast [8,9].

Eating breakfast compared with skipping the meal is associated with improved dietary quality [10,11]. Irregular breakfast consumption compared with regular consumption has been associated with e.g., cardiovascular diseases [12], type 2 diabetes [13,14,15], and body weight [16]. Further, breakfast consumption has a positive effect on cognitive performance in children and adolescents when compared with skipping the breakfast meal [17]. The literature on breakfast consumption has been summarized in a recent systematic review concluding that there is limited evidence of breakfast consumption being an important factor in combined weight and cardiometabolic risk management, but breakfast consumption can contribute to a healthier eating pattern [18].

In Denmark, few studies have been conducted on meal regularity and health and most studies on breakfast have described the breakfast consumption patterns according to age, gender, and socioeconomic status (SES) [9,19,20,21].

A multi-site randomized controlled trial including Danish participants tested the effectiveness of a recommendation to eat or skip breakfast on weight loss in adults. The authors concluded that the consumption of breakfast had no effect on weight loss [19]. The “Diet, Smoking, Alcohol and Exercise” (KRAM) study is the largest national health study of the Danish adult population, with 18,065 adults participating in a health examination [20]. The study examined the daily meal pattern of the participants and found that 82% of respondents reported eating breakfast every day and more women than men consumed breakfast regularly. Adolescents and young adults (18–24 years) had the most irregular breakfast patterns. The higher the education, the more regular breakfast patterns were seen. Those not eating breakfast every day had a higher intake of total fat, saturated fat and added sugar than those with more regular breakfast-eating patterns [20]. The World Health Organization’s (WHO) Study on Health Behaviour in School-aged Children (HBSC) has analyzed breakfast consumption in Danish children and adolescents. The latest study from 2014 showed that eight out of ten Danish 11-year-old children consumed breakfast every weekday. This proportion decreased in 15-year-olds where 66% of girls and 74% of boys had breakfast on a daily basis during the school week [21]. The regularity of meals and the intake of energy and nutrients in children and adults have been reported previously in the Danish National Survey of Diet and Physical Activity (DANSDA), and the results are similar to the HBSC study regarding regular breakfast patterns over time [8,9]. Breakfast habits in Denmark have recently been described and confirmed to have a high regularity of breakfast consumption among adults 18–74 years, as only 7% do not eat breakfast on a given day [22]. However, the 2016 report does not include data on nutrient intakes in relation to breakfast.

In the present study, DANSDA data are used to analyze breakfast consumption and association with the intake of key food groups, energy, and macro- and micronutrients. The analyses have been carried out in the aligned approach agreed in the International Breakfast Research Initiative (IBRI). IBRI was set up to study the nutritional impact of breakfast and provide evidence-based recommendations, primarily for a nutrient-based balanced breakfast [23]. The objectives of the present study are to describe the prevalence of breakfast consumption in Denmark, describe the contribution of breakfast to the daily nutrient intake and intake from key food groups and among breakfast consumers, compare energy, nutrient and food group intakes at breakfast by tertiles of daily dietary quality using the Nutrient Rich Food (NRF 9.3) Index. The prevalence of breakfast consumption and nutrient intake at breakfast are described for children, adolescents, younger and older adults, whereas two age groups (children/adolescents and adults) are used, when describing intakes across tertiles of NRF 9.3 scores.

## 2. Materials and Methods

### 2.1. Population

This study was based on data derived from the DANSDA 2011–2013, which is a nation-wide cross-sectional survey. Data were collected all year round during the survey period. The study population comprised of a simple random sample of 4 to 75-year-old children and adults retrieved from the Danish Civil Registration System. In comparison with census data from Statistics Denmark, the distribution of gender and age of the participants could be characterized as representative for the Danish population. However, individuals with a basic education were under-represented, and men and 19 to 54-year-olds were slightly under-represented [24]. In all, 3816 participants aged 6–75 years had complete dietary data and 3680 participants had 7 days of food records and were included in the present study. A total of 23 individuals did not eat breakfast in the week of dietary recording. Within the IBRI framework, it was decided that no lower limit for the energy content of breakfasts should be applied, thus all breakfasts are included in the analysis regardless of energy contribution.

The DANSDA survey has been conducted in accordance with the guidelines laid down in the Declaration of Helsinki and has been approved by the Danish Data Protection Agency. The Danish National Committee on Health Research Ethics has decided that, according to Danish Law, DANSDA does not require approval.

### 2.2. Intake of Energy, Nutrients, and Foods

The dietary intake was recorded every day for seven consecutive days in food diaries with pre-coded response categories, which included open-answer options. The food record was following a typical Danish meal pattern with breakfast, a morning snack, lunch, an afternoon snack, dinner, and evening snack. The amount of foods eaten was given in predefined household measures (cups, spoons, slices, etc.) or estimated from photos of up to six portion sizes. For food items not included in the pre-coded food record, the participants wrote the type of food and portion size eaten in open-answer categories. Participants also received a food diary booklet to take to school or to other places outside of the home on the days of food recording. Details about the method and calculation of intake of food and nutrients have been described elsewhere [25].

The intake of energy, nutrients, and food items was calculated for each individual using the software system GIES (version 1.000d; developed at the National Food Institute, Technical University of Denmark, Copenhagen, Demark), and the Danish Food Composition Databank [26]. In order to allow a focus solely on food-derived micronutrients, nutrient intakes from dietary supplements were not included in the calculation of nutrient intake and the current analysis. Food groups providing the major part of food intake at breakfast were chosen to describe the food intake at breakfast. The same food groups were chosen for children and adults, although the intake of some food groups are very low or zero in children (e.g., alcoholic beverages).

In the present study, the mean intake of energy, nutrients, and foods was calculated for breakfasts, as well as for the intake of the total day. The analysis in the study followed the harmonized approach defined within the IBRI consortium [23].

### 2.3. The Regularity of Breakfast Consumption

The regularity of breakfast was defined as follows:

- 5–7 breakfasts per week: Regular consumer
- 2–4 breakfasts per week: Irregular consumer
- Breakfast 0–1 day per week: Skipper

### 2.4. Calculation of Dietary Quality of the Diet by the NRF 9.3 Index

The dietary quality of the overall diet was calculated on the basis of a modified Nutrient Rich Foods (NRF) Index. The NRF Index calculates the nutrient density, defined as nutrients per calorie. The current NRF algorithms, based on 12 nutrients, can be applied to individual foods, meals or to the total diet [27]. The present version, known as NRF 9.3, was based on the sum of the percentage of daily values for nine nutrients to encourage protein, dietary fibre, vitamin A, vitamin C, vitamin E, calcium, iron, magnesium, and potassium, minus the sum of the percentage of maximum recommended values for three nutrients to limit saturated fat, total or added sugar, and sodium, with all daily values calculated per 2000 kcal and capped at 100% [28]. As used in the IBRI studies, the NRF 9.3 score reflects nutrient intakes (normalized to an intake of 2000 kcal) expressed as percentages of the national daily values for food labelling purposes. The IBRI consortium decided to replace vitamin E with vitamin D in the list of the nine nutrients to encourage, because vitamin D is a nutrient of public health concerns in many of the participating countries, including Denmark [24].

The algorithm for the NRF 9.3 Index subtracts the sum of the three nutrients to limit from the sum of the nine nutrients to encourage expressed as a multiple of 100:

(∑ sub-scores positive × 100) − (∑ sub-scores negative × 100)(1)

The sub-score was calculated for each nutrient. For the nutrients to encourage, sub-scores above 100 were truncated to 100 for that nutrient. For the nutrients to limit, if the sub-score was less than 100, then 0 was assigned to the sub-score. The maximum possible score was 900 points reflecting a diet where intakes per 2000 kcal were ≥ the daily values for the nine nutrients to encourage and were ≤ the daily values for the three nutrients to limit. Similarly, 600 points reflect a diet where intakes per 2000 kcal were ≥ the daily values for the nine nutrients to encourage and were ≥ the daily values for the three nutrients to limit, but there are numerous combinations of intake to achieve, e.g., 600 points. For the European countries participating in the IBRI studies, the NRF 9.3 scores were calculated on the basis of the EU reference daily values and maximum recommended values for nutrients based on a 2000-kcal diet (Table 1) [29]. Positive and negative nutrient sub-scores were calculated for both children and adults. For added sugar, an upper limit of 10% of energy intake was used as the nutrient reference value (NRV) according to the recommendation by the WHO [30]. The daily sodium intakes were converted to salt by applying the conversion factor of 2.5 [29] and the NRV was 6 g. The NRV for dietary fibre was defined as 25 g based on the European Food Safety Authority (EFSA) recommendation [31].

### 2.5. Statistical Analysis

SAS version 9.4 (SAS Institute Inc., Cary, NC, USA) was used for all statistical analyses. The significance level was chosen as *p* < 0.05. Model fit was checked by histograms and QQ (quantile-quantile) plots of the residuals. In order to meet the model assumption of normally distributed residuals, all macro- and micro-nutrient intakes were square root transformed and the food intakes were transformed using log10. Intakes of energy were not transformed.

Pearson Chi-square tests were used to test the difference in the regularity of breakfast consumption between gender and age groups, respectively. Non consumers of breakfast were included in the population for the Chi-square tests, but were excluded from all other statistics presented. Descriptive statistics for energy, nutrient, and food intake are given as the mean and standard deviation (SD). Education was chosen as a measure for SES as other research has shown education to be the key social variable of dietary habits in Denmark [32,33].

One-way analysis of variance (ANOVA) was used to compare the breakfast energy intake and all day energy intake between four age groups. Analysis of covariance (ANCOVA) adjusted for energy intake at breakfast was used to compare the nutrient intake at breakfast between the four age groups, while ANCOVA adjusted for daily energy intake was used to compare the daily nutrient intake between all age groups.

Tertiles of NRF 9.3 scores were calculated separately for children/adolescents (6–17 years) and adults (18–75 years). The upper tertile (T3) was indicative of the highest level of overall dietary quality. The two age groups were chosen to form the basis of the future work of IBRI developing two sets of nutritional guidelines for breakfast: one for children/adolescents and another for adults. A one-way ANOVA, and an ANCOVA adjusted for the daily energy intake and an ANCOVA adjusted for the daily energy intake and education was used to test the effect of tertile on nutrient and food intake (eaters only) at breakfast. The model check, as described above, was used to validate these models. The Tukey–Kramer post hoc test was used for multiple comparisons.

## 3. Results

### 3.1. The Regularity of Breakfast Consumption

**Figure 1:**
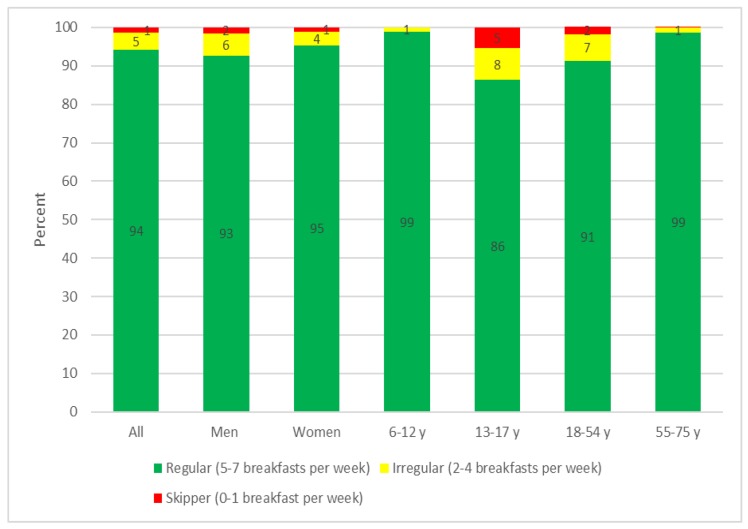
The proportion of skippers, irregular consumers, and regular consumers in the total Danish population according to gender and age (*n* = 3680).

Among the sample (*n* = 3680), the majority of the population (94.1%) were regular breakfast consumers, while 4.6% had an irregular pattern and 1.3% were classified as skippers (Figure 1). Slightly more women than men were regular breakfast consumers (*p* = 0.0023). Children (6–12 years) and older adults (55–75 years) had breakfast regularly (98.9% and 98.8% respectively) while adolescents (13–17 years) had an irregular pattern with 5.4% skipping breakfast and 8.3% eating breakfast 2–4 times a week. Among the adults (18–54 years), 7% were classified as irregular breakfast consumers. The chi-square test for homogeneity showed that the pattern of breakfast consumption was significantly different between the four age groups (*p* < 0.0001)

### 3.2. Daily and Breakfast Intake of Energy, Nutrients and Foods

Overall, 3657 participants consumed breakfast (476 children 6–12 years, 272 adolescents 13–17 years, 1791 younger adults 18–54 years, 1118 older adults 55–75 years) and were included in the analysis of nutrient and food intake. Table 2 and 3 show the unadjusted means and SD. The mean breakfast energy intake and daily energy intake were different (*p* = 0.0012 and *p* < 001, respectively) between age groups (Table 2). Differences between age groups were also found for the mean breakfast and daily intake of macronutrients.

The mean breakfast intake of micronutrients differed between the age groups (except folate and vitamin B_12_, both *p* > 0.05 (Table 3). The mean daily intake of micronutrients differed between age groups (except thiamin and riboflavin, both *p* > 0.10).

Descriptive figures of the mean intake of solid foods and beverages at breakfast according to the age groups are shown in Figures S1 and S2. Generally, the total intake of foods and beverages increased with increasing age. The intake of vegetables, cakes, soft drinks, and cordials (sweet fruit beverages with water) were low.

### 3.3. The Contribution of Breakfast to Daily Energy and Nutrient Intakes

**Figure 2:**
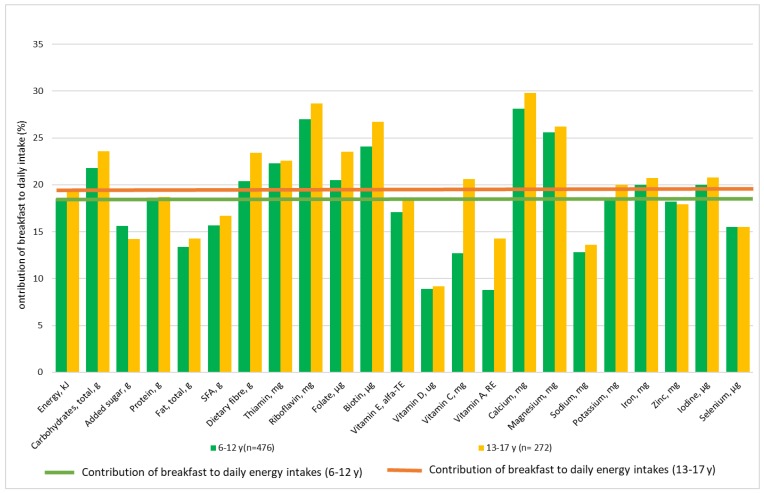
The contribution of breakfast (%) to the daily nutrient intakes in 6–12 and 13–17 year-old Danes.

**Figure 3:**
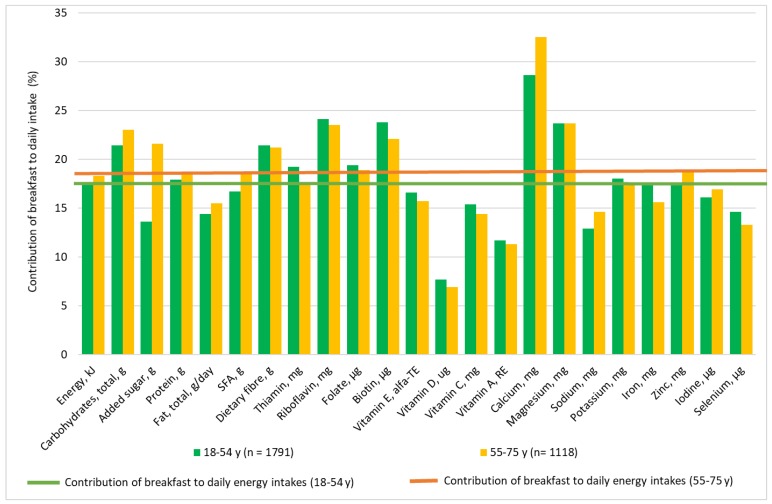
The contribution of breakfast (%) to the daily nutrient intakes in 18–54 and 55–75 year-old Danes.

The percentage contribution to daily nutrient intakes in children (6–12 years) and adolescents (13–17 years) is shown in Figure 2 and in younger (18–54 years) and older adults (55–75 years) in Figure 3. Breakfast provided 18–20% of daily energy intake. Among children and adolescents, breakfast contributed with a higher proportion of carbohydrates, dietary fibre, and several vitamins (thiamine, riboflavin, folate, and biotin) and minerals (calcium, magnesium, iron, and iodine) than energy to the daily diet. The contribution of added sugars, total fats, saturated fats, vitamins D and A, sodium, and selenium at breakfast was lower than the contribution of energy to the daily diet.

Among younger and older adults (Figure 3), breakfast contributed to a higher proportion of carbohydrates, dietary fibre, some vitamins (riboflavin, folate, and biotin) and minerals (calcium and magnesium) than energy to the daily diet. The contribution of total fats, vitamins C, D, E and A, sodium, iodine and selenium at breakfast was lower than the contribution of energy to the daily diet. The contribution of added sugars was higher than the contribution of energy to the daily diet among older adults.

### 3.4. Energy, Nutrients and Food Intakes at Breakfast by Tertiles of Daily Dietary Quality

Breakfast consumers were divided into tertiles of daily dietary quality based on the NRF 9.3 score. The population was split into two age groups, children/adolescents (6–17 years) and adults (18–75 years). Sociodemographic and lifestyle characteristics are shown in Table S1. Children/adolescents, who had a higher dietary quality, were more likely to be female and not be overweight/obese. Adults, who had a higher dietary quality, were more likely to be female, have an intention to eat healthily, were less likely to smoke daily or have a sedentary lifestyle.

A decrease in energy content of breakfast was seen in both age groups across tertiles of daily dietary quality as expressed by NRF 9.3 (Table 4). Intakes of added sugar, total fat and saturated fat (expressed as % breakfast energy intake) decreased significantly from the lowest to highest tertile of NRF 9.3 in children/adolescents and adults. The intake of protein (expressed as % breakfast energy intake) and dietary fibre increased in both age groups across tertiles of NRF 9.3.

The intake of most vitamins increased from the lowest to highest tertile of NRF 9.3 in children/adolescents and adults, except the intake of vitamin D adjusted for energy intake and education (both children/adolescents and adults) and vitamin E (children/adolescents) (Table 5). The intake of vitamin A decreased across the tertiles of NRF 9.3 in children/adolescents and adults.

The intake of calcium, iron, zinc, potassium, and magnesium increased from the lowest to highest tertile of NRF 9.3 in both age groups, whereas the intake of sodium decreased in both age groups.

In children/adolescents, the intake of breakfast cereals incl. oats and muesli and milk added to cereals was higher in the group with the highest dietary quality score (highest tertile of NRF 9.3). In contrast, the intake of fermented milk, non-whole grain wheat bread, fruits, tea and milk was higher in the lowest tertile of NRF 9.3 (Table 6). Otherwise, few significant differences across the tertiles were observed.

Among adults, the intake of breakfast cereals incl. oats and muesli, fruits and water increased across the tertiles of NRF 9.3, while non-whole grain bread and fats on bread decreased (Table 7). The intake of fermented dairy products was higher in the group with the highest dietary quality score (highest tertile of NRF 9.3). When comparing food intake in the groups with lowest and highest dietary quality, the study shows the higher intake of breakfast cereals, vegetables, fruits, dairy products and water, and lower non-whole grain bread in the group with highest dietary quality among adults.

## 4. Discussion

This study examined the regularity of breakfast consumption, energy, macro- and micronutrient and food group intakes at breakfast in Denmark. The relationship between nutrient and food intakes at breakfast and the overall dietary quality was also assessed within the Danish population. The relationship between nutrient intakes at breakfast and overall dietary quality in Denmark by using the Nutrient Rich Food Index 9.3 (NRF 9.3) has not been published previously.

The study shows that breakfast is eaten relatively frequently by children and adults in Denmark. This is in line with other Danish studies [20,21,22]. More women than men eat breakfast regularly and adolescents eat most irregularly. This also has been found in other Danish and international studies [20,21,34]. The present study shows that breakfast provides approximately 18–20% of the daily energy in all age groups. This finding is in line with previous studies of breakfast in Denmark [8,9], thus indicating a relatively staple contribution of breakfast to the total energy intake.

The study shows that commonly consumed foods at breakfast for Danish children, adolescents and adults are bread, breakfast cereals, and dairy products. Beverages consumed at breakfast are primarily water and juice (children and adolescents), coffee, water and juice (adults). Breakfast is a good source of carbohydrates, dietary fibre, B vitamins, calcium and magnesium, and is relatively low in fat, added sugar and sodium. The contribution of riboflavin, biotin, calcium, and magnesium from breakfast is 20–30% of the daily intake. This is due to the relatively high intake of dairy products and cereals at breakfast. In the Danish diet, the intake of cereals and dairy products (incl. cheese) accounts for 52% of the intake of riboflavin and 43% of the intake of magnesium, while dairy products (incl. cheese) account for 59% of the intake of calcium [24]. No data on the contribution of biotin are available in DANSDA. Breakfast contributes with approximately 10% of the daily intake of vitamin A and D. This reflects the low intake of foods rich in these vitamins at breakfast, but also illustrates the relatively low fortification practices in Denmark. Only salt is mandatorily fortified with iodine and despite the possibility to fortify foods voluntarily, few foods are fortified with nutrients. This might be due to a negative attitude among consumers towards foods fortified with nutrients [35,36].

Previous Danish analyses on the different dimensions of socioeconomic position (income, employment status, educational level etc.) on diet, health behavior, and obesity have shown education to be the key social variable of dietary habits in Denmark [37,38,39]. Education was therefore chosen as a measure for SES in this study. However, in the present study, the characteristics of participants showed no differences in education across the tertiles of NRF 9.3 scores. In relation to the measurement of dietary quality, a Danish diet quality index (DQI) has been used previously and the studies have shown a higher dietary quality with increasing education [33,39]. The Danish DQI is based on intake of macronutrients and food groups included in the Danish FBDG (total fats, saturated fats, fruits and vegetables, fish, and added sugar) [33,40]. In contrast to the Danish DQI, the NRF 9.3 Index is based to a larger extent on micronutrient intakes. As the content of micronutrients included in the NRF 9.3 is generally adequate in the average Danish diet [24], this might be part of the explanation for the lacking relationship between dietary quality and education, when using NRF 9.3 as a measure of dietary quality in Denmark. However, more analyses are needed to examine the differences between the two indices, how they rank different dietary intakes in the Danish diet, and the relationship between intake of food and the nutrients they provide.

In this study, other factors than education show relation to dietary quality. The low dietary quality group for adults comprises a higher proportion of males, individuals with a sedentary lifestyle and daily smokers and a lower proportion of individuals with the intention to eat healthily. Overall, these data suggest that individuals in the low dietary quality group have a less healthy lifestyle compared to the medium and high-quality dietary groups. The less healthy lifestyle includes less healthy dietary and breakfast habits due to a lower intake of breakfast cereals including milk and fruits and a higher intake of non-whole grain wheat bread, fats on bread and pastry/cakes/biscuits (adults). This together with other dietary factors is reflected in a lower NRF 9.3 score. These results are in line with previous analyses of DANSDA data showing that males and individuals with no intention to eat healthily consume a less healthy diet overall and that men compared with women have more unhealthy dietary habits and a higher intake of non-whole grain bread, cheese, red meat and fats and lower intake of fruits and vegetables [24,37,41]. This is reflected in the breakfast habits as well. When comparing food intake in the groups with lowest and highest dietary quality, the study showed a higher intake of breakfast cereals, vegetables, fruits, dairy products and water, and lower non-whole grain bread in the group with highest dietary quality among adults. Many of these foods are included in the Danish FBDG and the healthier food patterns at breakfast could be examined further in order to identify key food patterns in relation to nutrient intakes. Prior to the analysis of food patterns, it could be useful to disaggregate the food intake at breakfast into single food items in order to examine the intake of high and low-fat dairy products, the intake of high and low sugary breakfast cereals as well as the intake of food low in salt. Examining the intake of specific food items, will create a basis for the future work on the recommendations for a healthy breakfast in Denmark.

This study reveals good dietary practices among breakfast consumers and forms a basis for future dietary advice in Denmark. The low consumption of foods rich in sugar and sodium should be highlighted as a healthy dietary habit in public health campaigns. Additionally, the relatively high contribution of dietary fibre could be emphasized. However, keeping in mind that the overall intake of dietary fibre and whole grain is too low in Denmark [7,24] and with the study showing a high proportion of adults and especially children and adolescents eating non-whole grain bread for breakfast, a nutritional focus could be to substitute the non-whole grain foods with whole grain foods. The high intake of non-whole grain bread at breakfast among adolescents in combination with a lower intake of daily dietary fibre than the other age groups indicates a potential for raising the daily dietary fibre and whole grain intake among adolescents by increasing the whole grain intake at breakfast. In order to qualify advice on whole grain products at breakfast, it could be useful to evaluate how much of the recommended amount of whole grain is derived from breakfast. In addition to raising attention to whole grain cereals at breakfast, a focus could be on the intake of fruits and vegetables. The current study shows that a high proportion of Danes do not eat vegetables or fruits at breakfast. As Danes eat too little fruits and vegetables [24], the intake of fruits especially could be increased at breakfast. Regarding the intake of beverages, the good practice of drinking water at breakfast could be highlighted.

It has not been the aim of the current work to analyze the dietary quality among regular and irregular consumers of breakfast and in the analysis of dietary quality, regular and irregular consumers of breakfast were treated as one group. Even if breakfast is consumed regularly by the majority of the population, a future research project could be to analyze if regular and irregular consumers of breakfast have a different overall dietary quality.

Strengths of the study include the large nationally representative sample of the Danish population, and the assessment of nutrient and food group intakes at all meals based on seven days. Weighting factors were not applied within the data set to account for non-response bias. This may be seen as a limitation as adults with low educational level are underrepresented in DANSDA [24]. Therefore, we cannot exclude the possibility of systematic differences in breakfast and dietary habits between participants and non-participants as these groups differ in health behaviour [42,43]. The consequence may be the recording of healthier Danish breakfast habits than what is actually the case because higher educated individuals eat more healthily than lower educated. Still, adjustments were made for socioeconomic status in the statistical analyses.

Misreporting occurs in dietary surveys due to self-reported food intake. Previous research has shown that subjects with a high BMI, non-smokers, and subjects who eat healthily to avoid being overweight mostly under-report energy intake [44]. However, misreporting includes both over- and under-reporting and the exclusion of those misreporting may introduce selection and unknown biases into the data [45]. Instead of excluding implausible reporters from the current study, the energy intake was controlled for in the statistical analysis.

By expressing the contribution of nutrients from breakfast to daily energy and nutrient intakes, the study highlights that breakfast is an important meal providing many key nutrients. Together with the analysis of the impact of breakfast on the overall dietary quality and information on the percentage of consumers of different food groups, the results form a solid base for the future work of nutrient-based recommendations. The results can help Danish authorities in formulating more meal-based dietary guidelines.

## 5. Conclusions

The present study shows that breakfast is an important meal providing many key nutrients and it is high in dietary fibre and low in added sugars, total fat, saturated fat, and sodium. The analysis on the impact of breakfast on overall dietary quality form a solid base for future work of nutrient-based recommendations. The results can help Danish authorities in formulating more meal based dietary guidelines. The use of NRF 9.3 enables the comparison of dietary quality in different countries, but future research is needed to investigate the impact of using the NRF 9.3 index on a Danish diet to examine dietary quality. Additionally, further examination of why different dietary quality indices show different results regarding the relationship between education and dietary quality needs to be carried out.

On the basis of the results from the counties participating in IBRI, a set of guiding principles to derive nutrient guidelines for breakfast will be developed. The principles will be based on the nutritional profiles of breakfast among individuals in the NRF 9.3 upper tertile, taking into account the current public health interests in specific nutrients. Two sets of nutritional guidelines for breakfast will be developed: one for children and adolescents and another for adults.

The standardized approach used in IBRI can be applied on other meals in Denmark in order to form a basis for meal-based dietary guidelines, for example, lunch and dinner. This work can serve as a more evidence-based platform for authorities for the formulation of future meal-based dietary guidelines.

Overall, the study shows interesting results on both the food and nutrient level and more analyses can be carried out in order to qualify future meal-based guidelines.

## Figures and Tables

**Table 1:** The reference daily values and maximum recommended values for nutrients based on a 2000-kcal diet.

Nutrient	NRV ^1^	MRV ^2^
Protein (g)	50	-
Dietary fibre (g)	25	-
Vitamin A (ug)	800	-
Vitamin C (mg)	80	-
Vitamin D (ug)	5	-
Calcium (mg)	800	-
Iron (mg)	14	-
Potassium (mg)	2000	-
Magnesium (mg)	375	-
Saturated fat (E% fat)	-	10
Added sugar (E% fat)	-	10
Sodium (g)	-	6

^1^ NRV, nutrient reference value; ^2^ MRV; maximum recommended value.

**Table 2:** The intake of energy and macronutrients at breakfast and for the total day according to age in Danes

Age Group	All (6–75 years) *n* = 3657				Children (6–12 years) *n* = 476				Adolescents (13–17 years) *n* = 272				Younger Adults (18–54 years) *n* = 1791				Older Adults (55–75 years) *n* = 1118					
	Breakfast Intake		Daily Intake		Breakfast Intake		Daily Intake		Breakfast Intake		Daily Intake		Breakfast Intake		Daily Intake		Breakfast Intake		Daily Intake		*p* *	*p* **
	Mean	SD	Mean	SD	Mean	SD	Mean	SD	Mean	SD	Mean	SD	Mean	SD	Mean	SD	Mean	SD	Mean	SD	Breakfast Intake	Daily Intakes
Energy (kJ)	1736	806	9645	3066	1599	564	8655	1934	1759	820	9042	3365	1754	844	10029	3267	1761	824	9599	2932	0.0012	<0.0001
Carbohydrates (g)	59	28	266	87	59	22	269	62	64	31	273	104	58	28	273	91	58	28	251	82	<0.0001	<0.0001
Carbohydrates (E%)	56	12	47	6	60	8	51	5	60	11	50	6	55	12	47	6	54	12	45	6	<0.0001	<0.0001
Added sugars (g)	8	9	51	38	9	7	56	28	9	8	62	42	7	8	53	41	9	11	42	32	<0.0001	<0.0001
Added sugars (E%)	8	9	9	5	9	6	11	5	8	7	11	6	7	8	9	6	9	10	8	4	<0.0001	<0.0001
Proteins (g)	16	8	87	29	14	6	74	20	15	8	81	30	16	8	91	31	16	8	87	27	<0.0001	<0.0001
Proteins (E%)	16	5	16	3	15	3	15	2	15	5	16	3	16	6	16	3	16	5	17	3	<0.0001	<0.0001
Fat (g)	14	10	94	37	11	6	81	22	12	8	85	36	14	10	99	39	15	10	95	36	<0.0001	<0.0001
Fat (E%)	28	11	37	6	25	9	34	4	25	10	34	5	29	11	37	6	30	11	38	6	<0.0001	<0.0001
SFA (g)	7	5	38	16	5	3	33	10	6	4	34	16	7	5	3	17	7	5	38	16	<0.0001	<0.0001
SFA (E%)	13	6	15	3	12	5	14	2	12	6	14	3	13	6	15	3	14	7	15	3	<0.0001	<0.0001
Dietary fibre (g)	5	3	22	8	4	2	20	6	4	3	19	8	5	4	23	8	5	3	23	8	<0.0001	<0.0001

Abbreviation: SFA: Saturated fatty acids. *p* * for macronutrients adjusted for energy intake at breakfast *p* ** for macronutrients adjusted for daily energy intake. *p* < 0.05 considered significant. Values for macronutrients were Square root transformed prior to analysis. Mean and SD are based on absolute values of intake.

**Table 3:** The intake of micronutrients at breakfast and for the total day according to age in Danes.

Age Group	All (6–75 years) *n* = 3657				Children (6–12 years) *n* = 476				Adolescents (13–17 years) *n* = 272				Younger Adults (18–54 years) *n* = 1791				Older Adults (55–75 years) *n* = 1118					
	Breakfast Intake		Daily Intake		Breakfast Intake		Daily Intake		Breakfast Intake		Daily Intake		Breakfast Intake		Daily Intake		Breakfast Intake		Daily Intake		*p* *	*p* **
	Mean	SD	Mean	SD	Mean	SD	Mean	SD	Mean	SD	Mean	SD	Mean	SD	Mean	SD	Mean	SD	Mean	SD	Breakfast Intake	Daily Intake
Thiamin (mg)	0.3	0.1	1.4	0.5	0.3	0.1	1.3	0.4	0.3	0.2	1.3	0.5	0.3	0.2	1.4	0.5	0.2	0.1	1.4	0.5	<0.0001	0.4550
Riboflavin (mg)	0.4	0.2	1.8	0.7	0.4	0.2	1.6	0.5	0.5	0.3	1.7	0.7	0.4	0.2	1.8	0.7	0.4	0.2	1.8	0.6	<0.0001	0.1042
Niacin (NE)	5.1	2.5	33.0	11.7	3.4	1.5	23.8	6.9	4.0	2.0	27.2	10.6	5.3	2.6	34.9	12.1	5.8	2.4	35.2	10.6	<0.0001	<0.0001
B6 (mg)	0.2	0.1	1.6	0.5	0.2	0.1	1.3	0.4	0.2	0.1	1.4	0.5	0.2	0.1	1.7	0.6	0.2	0.1	1.6	0.5	<0.0001	<0.0001
Folate (ug)	67	36	340	129	59	25	288	86	67	35	286	109	69	40	355	133	67	34	351	132	0.0570	<0.0001
B12 (mg)	1.0	0.8	6.6	3.7	0.9	0.6	5.4	2.4	1.0	0.9	5.3	2.8	1.0	0.8	6.6	3.7	1.0	0.8	7.4	4.2	0.1320	<0.0001
Biotin (mg)	9	6	36	15	8	5	34	11	9	7	32	15	9	6	38	17	8	6	35	146	<0.0001	<0.0001
Vit E (alfa-TE)	1.5	1.4	8.9	4.0	1.3	1.2	7.6	2.6	1.3	1.3	7.2	3.4	1.5	1.5	9.3	4.3	1.4	1.4	9.1	4.0	0.0001	<0.0001
Vit D (ug)	0.3	0.9	4.4	4.2	0.2	0.2	2.7	2.1	0.3	0.3	2.9	2.7	0.4	0.5	4.4	3.9	0.4	1.5	5.7	5.1	0.0020	<0.0001
Vitamin C (mg)	17	20	112	57	13	15	103	52	19	23	94	51	18	20	114	58	17	21	117	58	0.0010	<0.0001
Vit A (RE)	148	236	1299	967	105	145	1189	706	139	211	973	737	155	244	1330	992	156	259	1376	1051	0.0030	<0.0001
Calcium (mg)	331	183	1113	413	295	141	1052	346	322	209	1081	441	332	186	1161	426	348	185	1071	403	0.0004	<0.0001
Iron (mg)	1.9	1.1	11.2	3.7	1.9	0.9	9.5	2.4	2.0	1.2	9.8	3.7	2.0	1.1	11.7	3.8	1.8	1.0	11.5	3.6	<0.0001	<0.0001
Zinc (mg)	2.2	1.1	12.0	4.2	1.8	0.9	10.1	2.8	2.0	1.2	11.2	4.4	2.2	1.2	12.6	4.4	2.3	1.2	12.1	3.9	<0.0001	<0.0001
Sodium (mg)	501	324	3726	127	413	206	3216	838	481	251	3534	1272	508	355	3941	1368	532	324	3646	1198	<0.0001	<0.0001
Iodine (ug)	42	26	247	142	47	24	233	119	49	32	235	128	42	26	259	157	40	24	236	129	<0.0001	0.0197
Potassium (mg)	619	280	3436	1097	504	212	2750	784	575	307	2875	1066	638	289	3556	1099	647	272	3673	1053	<0.0001	<0.0001
Magnesium (mg)	89	52	370	118	77	46	300	83	82	63	314	121	93	53	391	123	90	48	380	1072	<0.0001	<0.0001
Selenium (ug)	7.4	4.4	51.5	20.0	6.5	3.1	41.5	13.3	6.9	3.6	44.7	18.1	7.8	5.0	53.5	21.2	7.2	4.1	54.1	19.1	<0.0001	<0.0001

*p* * for micronutrients adjusted for energy intake at breakfast *p* ** for micronutrients adjusted for daily energy intake. *p* < 0.05 considered significant. Values for micronutrients were square root transformed prior to analysis. Mean and SD are based on absolute values of intake.

**Table 4:** The mean intake at breakfast of energy and micronutrients among breakfast consumers across the tertiles of the NRF 9.3 score according to age in Danes.

Age Group	Children/Adolescents 6–17 years (*n* = 748)									Adults 18–75 years (*n* = 2909)								
	Low Dietary Quality T_1_		Medium Dietary Quality T_2_		High Dietary Quality T_3_					Low Dietary Quality T_1_		Medium Dietary Quality T_2_		High Dietary Quality T_3_				
	Mean	SD	Mean	SD	Mean	SD	*p* *	*p* **	*p* ***	Mean	SD	Mean	SD	Mean	SD	*p* *	*p* **	*p* ***
NRF 9.3	538	66	644	20	729	41				571	66	685	30	774	95			
Energy (kJ)	1720	750	1632	628	1620	630	<0.0001	<0.0001	<0.0001	1861 ^a^	1021	1727 ^a^	741	1682 ^b^	701	<0.0001	<0.0001	0.0007
Carbohydrates (g)	60 ^b^	27	60 ^b^	25	62 ^a^	27	<0.0001	0.0008	0.0003	56 ^c^	30	57 ^b^	26	61 ^a^	28	<0.0001	<0.0001	<0.0001
Carbohydrates (%E)	58 ^c^	11	60 ^b^	9	63 ^a^	8	<0.0001	<0.0001	<0.0001	51 ^c^	14	54 ^b^	11	58 ^a^	11	<0.0001	<0.0001	<0.0001
Added sugars (g)	11 ^a^	9	9 ^b^	7	7 ^c^	7	<0.0001	<0.0001	<0.0001	10 ^a^	11	7 ^b^	8	7 ^a,b^	8	<0.0001	0.0049	0.0203
Added sugars (%E)	11 ^a^	8	9 ^b^	6	7 ^c^	5	<0.0001	<0.0001	<0.0001	10 ^a^	12	7 ^b^	7	7 ^b^	7	<0.0001	<.0001	<0.0001
Proteins (g)	14 ^b^	7	14 ^b^	6	15 ^a^	7	<0.0001	<0.0001	<0.0001	16 ^c^	10	16 ^b^	8	16 ^a^	7	<0.0001	<0.0001	<0.0001
Proteins (%E)	14 ^b^	4	14 ^b^	3	16 ^a^	4	<0.0001	<0.0001	<0.0001	15 ^c^	7	16 ^b^	4	17 ^a^	5	<0.0001	<0.0001	<0.0001
Fat (g)	13 ^a^	8	11 ^b^	6	9 ^c^	5	<0.0001	<0.0001	<0.0001	18 ^a^	13	14 ^b^	8	11 ^a^	7	<0.0001	<0.0001	<0.0001
Fat (%E)	28 ^a^	10	25 ^b^	9	21 ^c^	7	<0.0001	<0.0001	<0.0001	34 ^a^	12	29 ^b^	10	25 ^c^	9	<0.0001	<0.0001	<0.0001
SFA (g)	7 ^a^	4	5 ^b^	3	4 ^c^	2	<0.0001	<0.0001	<0.0001	9 ^a^	7	7 ^b^	4	5 ^c^	3	<0.0001	<0.0001	<0.0001
SFA (%E)	14 ^a^	6	12 ^b^	5	9 ^c^	4	<0.0001	<0.0001	<0.0001	17 ^a^	7	14 ^b^	6	10 ^c^	5	<0.0001	<0.0001	<0.0001
Dietary fibre (g)	4 ^c^	2	4 ^b^	2	5 ^a^	3	<0.0001	<0.0001	<0.0001	4 ^c^	2	5 ^b^	3	6 ^a^	3	<0.0001	<0.0001	<0.0001

Abbreviation: SFA: Saturated fatty acids *p* *. Unadjusted (ANOVA) *p* ** Adjusted for daily energy *p* *** adjusted for daily energy and education (ANCOVA). The Tukey–Kramer test for multiple comparisons was used. Different superscript letters indicate significantly different means for data adjusted for daily energy and education. *p* < 0.05 considered significant. Values for macronutrients were Square root transformed prior to analysis. Mean and SD are based on absolute values of intake.

**Table 5:** The mean intake at breakfast of micronutrients among breakfast consumers across the tertiles of the NRF 9.3 score according to age in Danes.

Age Group	Children/Adolescents 6–17 years (*n* = 748)									Adults 18–75 years (*n* = 2909)								
	Low Dietary Quality T_1_		Medium Dietary Quality T_2_		High Dietary Quality T_3_					Low Dietary Quality T_1_		Medium Dietary Quality T_2_		High Dietary Quality T_3_				
	Mean	SD	Mean	SD	Mean	SD	*p* *	*p* **	*p* ***	Mean	SD	Mean	SD	Mean	SD	*p* *	*p* **	*p* ***
Thiamin (mg)	0.27 ^c^	0.13	0.28 ^b^	0.13	0.32 ^a^	0.16	<0.0001	<0.0001	<0.0001	0.23 ^c^	0.14	0.26 ^b^	0.14	0.29 ^a^	0.14	<0.0001	<0.0001	<0.0001
Riboflavin (mg)	0.44 ^a^	0.25	0.44 ^b^	0.22	0.49 ^a^	0.23	<0.0001	<0.0001	<0.0001	0.40 ^c^	0.26	0.43 ^b^	0.22	0.45 ^a^	0.21	<0.0001	<0.0001	<0.0001
Niacin (NE)	3.5 ^b^	1.8	3.4 ^a^	1.5	4.0 ^a^	1.7	<0.0001	<0.0001	<0.0001	5.4 ^c^	2.9	5.6 ^b^	2.4	5.6 ^a^	2.2	<0.0001	<0.0001	<0.0001
B6 (mg)	0.20 ^b^	0.11	0.21 ^a^	0.10	0.25 ^a^	0.10	<0.0001	<0.0001	<0.0001	0.19 ^c^	0.12	0.23 ^b^	0.13	0.25 ^a^	0.13	<0.0001	<0.0001	<0.0001
Folate (ug)	61.5 ^b^	32.6	60.6 ^a^	27.2	63.9 ^a^	27.3	<0.0001	0.0036	0.0008	63.3 ^c^	37.1	68.5 ^b^	35.8	72.2 ^a^	39.9	<0.0001	<0.0001	<0.0001
B12 (mg)	0.91 ^b^	0.76	0.89 ^a^	0.67	1.08 ^a^	0.66	<0.0001	<0.0001	<0.0001	1.01 ^b^	0.97	1.03 ^a^	0.75	1.01 ^a^	0.64	<0.0001	<0.0001	<0.0001
Biotin (mg)	6.5 ^c^	4.4	7.6 ^b^	4.7	10.6 ^a^	6.7	<0.0001	<0.0001	<0.0001	7.0 ^c^	5.6	8.4 ^b^	5.6	10.1 ^a^	6.5	<0.0001	<0.0001	<0.0001
Vit E (alfa-TE)	1.59 ^a^	1.66	1.26 ^b^	1.19	1.08 ^c^	0.79	<0.0001	0.0058	0.0320	1.44 ^b^	1.33	1.40 ^c^	1.30	1.65 ^a^	1.70	<0.0001	<0.0001	<0.0001
Vit D (ug)	0.28 ^a^	0.27	0.23 ^a,b^	0.16	0.25 ^b^	0.19	<0.0001	0.1986	0.1432	0.34 ^b^	0.38	0.33 ^b^	0.78	0.41 ^a^	1.54	<0.0001	0.0592	0.1035
Vitamin C (mg)	15.2 ^b^	21.7	14.9 ^a,b^	17.0	16.0 ^a^	15.7	<0.0001	0.0253	0.0041	13.0 ^c^	17.0	17.7 ^b^	20.0	21.4 ^a^	23.5	<0.0001	<0.0001	<0.0001
Vit A (RE)	136	197	113	151	102	166	<0.0001	0.0100	0.0070	201 ^a^	296	154 ^b^	220	112 ^c^	217	<0.0001	<0.0001	<0.0001
Calcium (mg)	284 ^c^	182	293 ^b^	152	339 ^a^	169	<0.0001	<0.0001	<0.0001	313 ^c^	211	344 ^b^	178	358 ^a^	161	<0.0001	<0.0001	<0.0001
Iron (mg)	1.76 ^c^	0.86	1.88 ^b^	0.94	2.19 ^a^	1.16	<0.0001	<0.0001	<0.0001	1.74 ^c^	1.04	1.89 ^b^	1.00	2.18 ^a^	1.17	<0.0001	<0.0001	<0.0001
Zinc (mg)	1.70 ^c^	0.92	1.80 ^b^	0.88	2.18 ^a^	1.12	<0.0001	<0.0001	<0.0001	2.07 ^c^	1.29	2.25 ^b^	1.11	2.39 ^a^	1.08	<0.0001	<0.0001	<0.0001
Sodium (mg)	500 ^a^	255	427 ^b^	203	386 ^b^	201	<0.0001	<0.0001	0.0010	616 ^a^	421	528 ^a^	313	409 ^b^	239	<0.0001	<0.0001	<0.0001
Iodine (ug)	46.2 ^b^	29.3	45.7 ^b^	24.7	50.6 ^a^	25.7	<0.0001	0.0005	0.0001	38.1 ^c^	27.8	41.4 ^b^	24.4	43.7 ^a^	22.3	<0.0001	<0.0001	<0.0001
Potassium (mg)	482 ^c^	271	507 ^b^	217	600 ^a^	253	<0.0001	<0.0001	<0.0001	556 ^c^	262	644 ^b^	270	726 ^a^	289	<0.0001	<0.0001	<0.0001
Magnesium (mg)	63.4 ^c^	37.9	74.4 ^b^	44.5	99.0 ^a^	64.9	<0.0001	<0.0001	<0.0001	74.0 ^c^	39.8	90.1 ^b^	47.2	110.7 ^a^	57.4	<0.0001	<0.0001	<0.0001
Selenium (ug)	6.54 ^b^	3.72	6.24 ^b^	2.90	7.10 ^a^	3.21	<0.0001	<0.0001	<0.0001	7.49 ^c^	5.37	7.54 ^b^	4.21	7.74 ^a^	4.37	<0.0001	<0.0001	<0.0001

*p* * Unadjusted (ANOVA) *p* ** Adjusted for daily energy *p* *** adjusted for daily energy and education (ANCOVA). The Tukey–Kramer test for multiple comparisons was used. Different superscript letters indicate significantly different means for data adjusted for daily energy and education. *p* < 0.05 considered significant. Values for micronutrients were Square root transformed prior to analysis. Mean and SD are based on absolute values of intake.

**Table 6:** The mean intake at breakfast of selected food groups (eaters only) among breakfast consumers across the tertiles of the NRF 9.3 score among Danish children.

Age Group	Children/Adolescents 6–17 years (*n* = 748)											
	Low Dietary Quality T_1_			Medium Dietary Quality T_2_			High Dietary QualityT_3_					
	% Consumers (*n*)	Mean	SD	% Consumers (*n*)	Mean	SD	% Consumers (*n*)	Mean	SD	*p* *	*p* **	p***
Breakfast cereals incl. oats & muesli (g)	58 (143)	22 ^b^	19	73 (183)	20 ^b^	24	82 (204)	37 ^a^	33	<0.0001	<0.0001	<0.0001
Porridge (oats, rice etc.) (g)	10 (25)	68	67	13 (33)	75	69	18 (42)	84	75	0.4253	0.4238	0.3771
Milk for breakfast cereals (g)	36 (90)	75 ^b^	48	50 (125)	90 ^b^	62	58 (145)	105 ^a^	65	0.0014	0.0010	0.0100
Fermented dairy products (g)	25 (63)	84 ^a^	70	37 (92)	72 ^b^	63	29 (72)	63 ^b^	49	0.0722	0.1184	0.0869
Non-whole grain wheat bread (g)	80 (200)	38 ^a^	31	68 (169)	34 ^a^	27	60 (151)	23 ^b^	20	<0.0001	<0.0001	0.0002
Whole grain wheat bread (g)	39 (98)	28	23	46 (115)	29	25	43 (108)	27	23	0.7045	0.8552	0.9845
Whole grain rye bread (g)	16 (41)	19	17	17 (42)	16	16	24 (59)	19	19	0.7540	0.6835	0.7101
Pastry, cakes, biscuits (g)	18 (44)	17	18	13(33)	13	11	12(29)	12	7	0.5758	0.6673	0.9216
Fats on bread (g)	73 (183)	7^a^	6	73(182)	6 ^a^	4	58(146)	4 ^b^	4	<0.0001	<0.0001	<0.0001
Cold cuts, cheese, egg on bread (g)	89 (222)	22	20	86(214)	17	14	81(202)	16	15	0.0026	0.0326	0.2876
Vegetables, rich in dietary fibre (g)	1 (2)	16	8	5(12)	9	12	4(11)	14	13	0.3371	0.4983	0.3407
Vegetables, low in dietary fibre (g)	3 (8)	19	19	5(12)	12	11	6(15)	17	16	0.8888	0.9426	0.2989
Fruits (g)	22 (54)	45 ^a^	88	35(87)	33 ^a,b^	36	54(134)	41^b^	52	0.2760	0.1162	0.0787
Beverages total (g)	97 (242)	226 ^b^	134	98 (245)	199 ^b^	122	98 (246)	219 ^a^	132	0.0560	0.0658	0.0190
Coffee (g)	4 (10)	188	170	4 (10)	110	80	5 (12)	113	67	0.7356	0.7396	0.6723
Tea (g)	10 (26)	99 ^a^	68	8 (21)	81 ^b^	45	13 (33)	78 ^b^	69	0.1310	0.1586	0.0819
Water (g)	38(93)	106	81	45(111)	109	103	48 (121)	105	104	0.8085	0.6899	0.8720
Milk (incl. milk with chocolate flavor/fruits) (g)	71 (176)	153 ^a,b^	125	74 (185)	122 ^b^	101	74 (186)	147 ^a^	101	0.0287	0.0263	0.0019
Juice (g)	41 (103)	99	87	48 (119)	83	81	46 (116)	75	63	0.0914	0.1790	0.6084
Cordial (g)	8 (19)	66	45	4 (11)	53	22	2 (6)	76	49	0.7231	0.7352	0.4738
Cordial light (g)	3 (8)	80	84	3 (8)	44	30	1 (3)	57	49	0.3943	0.4086	0.1834
Carbonated soft drinks sugar sweetened (g)	6 (14)	47	29	1 (2)	32	15	-	n/a	n/a	0.7012	0.7326	0.5374
Carbonated soft drinks sugar light (g)	0	n/a	n/a	− (1)	29	0	− (1)	21	n/a	<0.0001	n/a	n/a
Beer (g)	0	n/a	n/a	0	n/a	n/a	0	n/a	n/a	n/a	n/a	n/a
Wine/spirits (g)	0	n/a	n/a	0	n/a	n/a	0	n/a	n/a	n/a	n/a	n/a

Under 1%. n/a: not applicable. *p* *. Unadjusted (ANOVA) *p* ** Adjusted for daily energy *p* *** adjusted for daily energy and education (ANCOVA). The Tukey–Kramer test for multiple comparisons was used. Different superscript letters indicate significantly different means for data adjusted for daily energy and education. *p* < 0.05 considered significant. Values for food intake were Log (10) transformed prior to analysis. Mean and SD are based on absolute values of intake. No statistical tests were carried out for % of consumers.

**Table 7:** The mean intake at breakfast of selected food groups (eaters only) among breakfast consumers across the tertiles of the NRF 9.3 score among Danish adults.

Age Group	Adults 18–75 years (*n* = 2909)											
	Low Dietary Quality T_1_			Medium Dietary Quality T_2_			High Dietary Quality T_3_					
	% Consumers (*n*)	Mean	SD	% Consumers (*n*)	Mean	SD	% Consumers (*n*)	Mean	SD	*p* *	*p* **	*p* ***
Breakfast cereals incl. oats & muesli (g)	38 (367)	27 ^c^	23	57 (551)	31 ^b^	25	71 (688)	37 ^a^	29	<0.0001	<0.0001	<0.0001
Porridge (oats, rice etc.) (g)	8 (75)	81	67	11 (108)	90	91	15 (145)	98	94	0.6462	0.3394	0.4779
Milk for breakfast cereals (g)	22 (217)	89 ^b^	50	32 (309)	94 ^a,b^	51	40 (383)	102 ^a^	53	0.0161	0.0009	0.0110
Fermented dairy products (g)	23 (220)	96 ^b^	88	34 (331)	100 ^b^	81	42 (406)	115 ^a^	85	0.0019	<0.0001	<0.0001
Non-whole grain wheat bread (g)	73 (708)	41^a^	34	62 (600)	27 ^b^	21	47 (459	20 ^c^	17	<0.0001	<0.0001	<0.0001
Whole grain wheat bread (g)	35 (337)	29 ^a,b^	22	45 (439)	29 ^a,b^	22	46 (444)	26^b^	22	0.0003	0.0601	0.1251
Whole grain rye bread (g)	39 (373)	23	18	50 (42)	16	16	46 (59)	19	19	0.0523	0.3660	0.6989
Pastry, cakes, biscuits (g)	14 (137)	17 ^a^	16	11 (104)	13 ^a,b^	10	9 (84)	10 ^b^	9	0.0003	0.0042	0.0396
Fats on bread (g)	75 (728)	11 ^a^	9	64 (620)	7 ^b^	6	43 (419)	5 ^c^	5	<0.0001	<0.0001	<0.0001
Cold cuts, cheese, egg on bread (g)	86 (839)	43 ^a^	37	88 (854)	37 ^a,b^	32	82 (798)	33 ^b^	32	<0.0001	0.0039	0.0292
Vegetables, rich in dietary fibre (g)	3 (26)	15 ^b^	14	5 (50)	30 ^a^	54	6 (58)	28 ^a^	42	0.1456	0.1462	0.0852
Vegetables, low in dietary fibre (g)	6 (58)	18	27	7 (63)	22	27	7 (65)	21	21	0.3237	0.1755	0.4446
Fruits (g)	29 (284)	40^c^	41	49 (477)	54 ^b^	57	66 (643)	67^a^	82	<0.0001	<0.0001	<0.0001
Beverages total (g)	98 (954)	439 ^c^	228	99 (960)	475 ^b^	221	98 (963)	497 ^a^	229	<0.0001	<0.0001	<0.0001
Coffee (g)	70 (674)	113^c^	67	75 (723)	291 ^a^	167	75 (724)	264 ^b^	158	<0.0001	<0.0001	<0.0001
Tea (g)	15 (141)	175	144	22 (213)	195	172	27 (260)	201	169	0.4072	0.1432	0.2605
Water (g)	51 (498)	170 ^c^	128	63 (611)	196 ^b^	139	73 (712)	224^a^	149	<0.0001	<0.0001	<0.0001
Milk (incl. milk with chocolate flavor/fruits) (g)	37 (256)	140	120	37 (358)	124	100	33 (322)	118	95	0.0567	0.3336	0.5149
Juice (g)	29 (283)	95	71	36 (348)	98	73	36 (349)	93	68	0.7467	0.9310	0.9168
Cordial (g)	5 (45)	88	74	3 (31)	68	49	2 (23)	74	80	0.2370	0.2774	0.2553
Cordial light (g)	3(24)	99	72	2 (21)	80	63	1 (12)	91	61	0.6213	0.6174	0.4768
Carbonated soft drinks sugar sweetened (g)	4 (40)	128 ^a^	129	1 (13)	47 ^b^	34	1 (5)	77 ^a,b^	101	0.0037	0.0103	0.0237
Carbonated soft drinks sugar light (g)	2 (17)	142	187	1 (11)	55	27	− (3)	107	126	0.4415	0.6744	0.3888
Beer (g)	− (2)	79	44	− (4)	159	135	0	n/a	n/a	0.8205	0.4893	0.3807
Wine/spirits (g)	3 (30)	17	22	3 (30)	10	7	4 (42)	7	4	0.0012	0.0022	0.0057

Under 1%. Abbreviations: n/a not applicable. *p* *. Unadjusted (ANOVA) *p* ** Adjusted for daily energy *p* *** adjusted for daily energy and education (ANCOVA). The Tukey–Kramer test for multiple comparisons was used. Different superscript letters indicate significantly different means for data adjusted for daily energy and education. *p* < 0.05 considered significant. Values for food intake were Log (10) transformed prior to analysis. Mean and SD are based on absolute values of intake. No statistical tests were carried out for % of consumers.

## Supplementary Materials

The following are available online at http://www.mdpi.com/2072-6643/10/8/1085/s1, Table S1: Sociodemographic and lifestyle characteristics of children and adults breakfast consumers across tertiles of NRF 9.3. Figure S1: Mean intake of solid foods (g) at breakfast according to age group. Figure S2: Mean intake of beverages (g) at breakfast according to age group.
