# Supplementary material for: Breakfast in Denmark. Prevalence of Consumption, Intake of Foods, Nutrients and Dietary Quality. A Study from the International Breakfast Research Initiative
Source: Nutrients. 2018 Aug 14;10(8):1085. doi: 10.3390/nu10081085 (PMC6116167; doi:10.3390/nu10081085)
Supplement: Supplementary file 1 [file nutrients-10-01085-s001.docx]

Supplement:

Governing principles document

**Table S1.** Socio demographic and lifestyle characteristics of Danish children and adult breakfast consumers across the tertiles of NRF 9.3.

| **Age group** | **Children 6**–**17 years (*n* = 748)** | | | | **Adults 18**–**75 years (*n* = 2909)** | | |  |
| --- | --- | --- | --- | --- | --- | --- | --- | --- |
|  | **Low Dietary Quality**  **T_1_** | **Medium Dietary Quality**  **T_2_** | **High Dietary Quality**  **T_3_** | | **Low Dietary quality**  **T_1_** | **Medium Dietary Quality**  **T_2_** | **High Dietary Quality**  **T_3_** |  |
| **Characteristic** | **Mean** | **Mean** | **Mean** | ***p*** | **Mean** | **Mean** | **Mean** | ***p*** |
| Mean age ± SD | 11.0±2.9 | 10.7±13.5 | 9.9±12.1 |  | 46.1±27.4 | 49.4±16.9 | 53.7±9.2 |  |
| Sex (% male) | 53.3 | 56.5 | 42.3 | <.001 | 60.7 | 48.9 | 36.0 | <0.001 |
| Sex (% female) | 46.7 | 43.5 | 57.7 | <.001 | 39.3 | 51.1 | 64.0 | <0.001 |
| Education (Basic/vocational) (%) # | 46.7 | 43.5 | 46.1 | 0.439 | 56.8 | 56.9 | 59.6 | 0.999 |
| Overweight/Obese (%) * | 23.3 | 18.5 | 16.7 | 0.046 | 59.8 | 58.7 | 56.4 | 0.381 |
| Sedentary lifestyle (%) | n/a | n/a | n/a | n/a | 11.3 | 6.0 | 3.6 | <0.001 |
| Daily smoker (%) | n/a | n/a | n/a | n/a | 27.3 | 16.9 | 9.1 | <0.001 |
| Intention to eat healthily (yes) (%) | n/a | n/a | n/a | n/a | 63.1 | 86.2 | 94.1 | <0.001 |

Abbreviations: n/a = not applicable. # For children, only parental education for 6–14 year-olds is included. * Based on measured height and weight. BMI Cole used for 6–17 years. Tested using Chi-square test (*p* < 0.05).

**Figure S1.** The mean intake of solid foods (g) at breakfast according to age group in Danes.

**Figure S2.** The mean intake of beverages (g) at breakfast according to age group in Danes.
